# Safety and EEG data quality of concurrent high-density EEG and high-speed fMRI at 3 Tesla

**DOI:** 10.1371/journal.pone.0178409

**Published:** 2017-05-26

**Authors:** Mette Thrane Foged, Ulrich Lindberg, Kishore Vakamudi, Henrik B. W. Larsson, Lars H. Pinborg, Troels W. Kjær, Martin Fabricius, Claus Svarer, Brice Ozenne, Carsten Thomsen, Sándor Beniczky, Olaf B. Paulson, Stefan Posse

**Affiliations:** 1 Neurobiology Research Unit, Department of Neurology, Rigshospitalet, Copenhagen, Denmark; 2 Department of Clinical Medicine, University of Copenhagen, Denmark; 3 Functional Imaging Unit, Department of Clinical Physiology, Nuclear Medicine and PET, Rigshospitalet, Copenhagen, Denmark; 4 Department of Neurology, University of New Mexico, Albuquerque, NM, United States of America; 5 Department of Physics and Astronomy, University of New Mexico, Albuquerque, NM, United States of America; 6 Department of Clinical Neurophysiology, Rigshospitalet, Copenhagen, Denmark; 7 Department of Biostatistics, University of Copenhagen, Denmark; 8 Department of Radiology, Rigshospitalet, Copenhagen, Denmark; 9 Department of Clinical Neurophysiology, Danish Epilepsy Centre, Dianalund, Denmark; 10 Department of Clinical Neurophysiology, Aarhus University, Aarhus, Denmark; 11 Department of Electrical and Computer Engineering, University of New Mexico, Albuquerque, NM, United States of America; University of Zurich, SWITZERLAND

## Abstract

**Purpose:**

Concurrent EEG and fMRI is increasingly used to characterize the spatial-temporal dynamics of brain activity. However, most studies to date have been limited to conventional echo-planar imaging (EPI). There is considerable interest in integrating recently developed high-speed fMRI methods with high-density EEG to increase temporal resolution and sensitivity for task-based and resting state fMRI, and for detecting interictal spikes in epilepsy. In the present study using concurrent high-density EEG and recently developed high-speed fMRI methods, we investigate safety of radiofrequency (RF) related heating, the effect of EEG on cortical signal-to-noise ratio (SNR) in fMRI, and assess EEG data quality.

**Materials and methods:**

The study compared EPI, multi-echo EPI, multi-band EPI and multi-slab echo-volumar imaging pulse sequences, using clinical 3 Tesla MR scanners from two different vendors that were equipped with 64- and 256-channel MR-compatible EEG systems, respectively, and receive only array head coils. Data were collected in 11 healthy controls (3 males, age range 18–70 years) and 13 patients with epilepsy (8 males, age range 21–67 years). Three of the healthy controls were scanned with the 256-channel EEG system, the other subjects were scanned with the 64-channel EEG system. Scalp surface temperature, SNR in occipital cortex and head movement were measured with and without the EEG cap. The degree of artifacts and the ability to identify background activity was assessed by visual analysis by a trained expert in the 64 channel EEG data (7 healthy controls, 13 patients).

**Results:**

RF induced heating at the surface of the EEG electrodes during a 30-minute scan period with stable temperature prior to scanning did not exceed 1.0° C with either EEG system and any of the pulse sequences used in this study. There was no significant decrease in cortical SNR due to the presence of the EEG cap (p > 0.05). No significant differences in the visually analyzed EEG data quality were found between EEG recorded during high-speed fMRI and during conventional EPI (p = 0.78). Residual ballistocardiographic artifacts resulted in 58% of EEG data being rated as poor quality.

**Conclusion:**

This study demonstrates that high-density EEG can be safely implemented in conjunction with high-speed fMRI and that high-speed fMRI does not adversely affect EEG data quality. However, the deterioration of the EEG quality due to residual ballistocardiographic artifacts remains a significant constraint for routine clinical applications of concurrent EEG-fMRI.

## Introduction

Simultaneous electroencephalography (EEG) and functional magnetic resonance imaging (fMRI) have in recent years emerged as a new tool in brain research. EEG-fMRI combines the high temporal resolution of EEG in milliseconds range with the high spatial resolution of fMRI in the mm^3^ range. This might enable improved localization of the sources of the electrical events based on the associated blood oxygenation level dependent (BOLD) response. Concurrent acquisition of fMRI and EEG has also emerged as a new tool for localizing the epileptogenic zone. In medically intractable epilepsy patients, removal of the epileptogenic tissue is a well-documented treatment in cases where the epileptogenic focus can be localized [1] [2] [3], and here, EEG-fMRI offers the potential for improved spatial localization of the epileptogenic zone. However, whether fMRI is capable of mapping the onset of the epileptic discharge and its spread, and how BOLD signal changes are related to the epileptic activity is an area of active research [4] [5]. The use of concurrent EEG-fMRI may have even wider application in neurobiological research, e.g., during fMRI recording of functional activation and evoked potentials as well as for MR sleep recording.

The acquisition of high quality EEG data inside the MRI scanner environment is challenging and much effort has been spent on deconvolving gradient, motion and ballistocardiographic (BCG) pulse artifacts [6] [7]. EEG recordings inside the MR scanner are prone to strong artifacts including gradient artifacts due to the currents induced by gradient switching, to head movements, and to ballistocardiographic (BCG) pulse artifacts. The BCG pulse artifacts are thought to be primarily caused by small movements of the body and the electrodes due to cardiac pulsation in arteries and to a smaller extent due to the Hall effect. Furthermore, the supine position inside the MR scanner may be associated with artifacts in the signals of the posterior electrodes during head movement. The sensitivity of measuring background activity, such as the alpha rhythm (8–13 Hz), in the EEG data has been used in studies for assessing the performance of artifact reduction algorithms [6].

The impact on fMRI data quality with concurrent use of EEG equipment has been investigated in a number of studies, which reported increased inhomogeneity of the static magnetic field in the vicinity of the electrodes causing decreases in cortical signal-to-noise ratio (SNR), but no significant effect on the sensitivity of detecting task-based fMRI signal changes and resting state networks [8] [9] [10] [11].

Recent advances in increasing the temporal resolution of fMRI using high-speed acquisition methods that enable un-aliased sampling of physiological signal fluctuation (conventional fMRI methods suffer from cardiac-related aliased signal fluctuations that interfere with activation-related signal changes) have considerably increased sensitivity for mapping task-based activation and functional connectivity, as well as for detecting dynamic changes in connectivity over time [12] [13] [14]. A concurrent EEG-fMRI study in patients with epilepsy demonstrated that ultra-fast fMRI using magnetic resonance encephalography (MREG) increased sensitivity for localizing interictal spikes when compared with conventional EPI [15]. However, MREG has limited spatial resolution and requires long image reconstruction times. RF power requirements are comparable to EPI. Simultaneous multi-slice (multi-band) EPI (MB-EPI) is now widely used for resting state fMRI and represents a promising methodology for concurrent EEG-fMRI, but increased RF power requirements of multi-band RF pulses are a safety concern. Multi-slab echo volumar imaging (MS-EVI), a 3D encoded high-speed fMRI technique that has also been shown to increase fMRI sensitivity for mapping task-based and resting state connectivity in healthy controls and patients with brain lesions [13] [16], has less stringent radiofrequency (RF) power requirements than MB-EPI.

A key concern is RF related heating of the EEG leads and electrodes, which depends on many MR acquisition related factors. RF heating has been shown to be mainly related to the specific absorption rate (SAR) of RF energy [8] [17]. Low SAR is considered a core safety requirement for EEG-fMRI. A confounding issue is that the RF pulse shapes for slice selection and lipid suppression that are used in different pulse sequences are vendor specific. Further, the EEG wire layout inside the head RF coil depends on the coil geometry and the head size relative to the coil inner diameter, leading to increases in average RF power levels and SAR. The safety of concurrent EEG-fMRI has been characterized at field strengths up to 7 Tesla [8] [17]. However, most of these studies have focused on conventional echo-planar imaging (EPI) with temporal resolution of 2–3 seconds. As stated above, MB-EPI increases SAR, whereas MREG and MEVI have low SAR. A second concern is fast gradient switching rates with high-speed fMRI increase the potential for gradient artifacts. There is thus an urgent need to assess the safety and data quality of concurrent EEG-fMRI using recently developed high-speed fMRI acquisition methods.

Our present study investigated RF heating in concurrent EEG and high-speed fMRI by comparing three different fMRI pulse sequences (EPI, MB-EPI and MS-EVI) in healthy controls and patients with epilepsy using two different 3 Tesla MRI scanners from 2 different vendors equipped with a 64- and 256-channel MR-compatible EEG system, respectively. RF heating was examined by direct multi-point temperature measurements. The effect of the EEG cap on the signal-to-noise ratio was evaluated in occipital cortex. EEG data quality was assessed using the 64-channel EEG system. Here we investigated to which extent residual gradient artifacts due to the choice of the fMRI pulse sequence (EPI and MEPI compared to MB-EPI and MS-EVI), BCG artifacts and patient movement impact the data quality of EEG and ongoing activity recorded inside an MR scanner.

## Materials and methods

### Subjects

The project was approved by the Ethics Committee of the Capital Region of Copenhagen Denmark (H-KA-20060151 and H-2-2013-038). All subjects (controls and patients) gave written informed consent before participation. The controls were recruited from an online database and by word of mouth. Recordings consisted of MR scans, EEG recordings and temperature measurements between the skin and the EEG electrodes.

### Controls

A total of 11 healthy controls were studied: 8 subjects were scanned using the Philips Achieva (3 males, age between 18 and 70 years), and 3 subjects using the Siemens Prisma scanner (3 females, age between 20 and 26 years). In the subjects studied on the Phillips scanner the EEG quality was assessed in all except 1 and systematic temperature measurements in 3. In all subjects studied on the Siemens scanner the temperature was systematically investigated.

### Patients

A total of 13 patients were scanned (8 males, age between 21 and 67) using the Philips Achieva scanner and EEG quality was assessed. Judged by EEG, MR and semiology 7 had temporal lobe epilepsy, 1 had probable temporal lobe epilepsy, 2 had frontal lobe epilepsy, 1 had epilepsy arising from the occipital lobes, 1 had idiopathic generalized epilepsy, and 1 had epilepsy arising from the central region. All patients were on anti-epileptic medication based on best clinical practice. The patient inclusion criteria were: adults (> 18 years), with epilepsy and a history of frequent interictal spikes or sharp waves (aiming for > 5 per 1/2 hour) with high amplitude (aiming for > 70μV), who could cooperate with the examination.

### Equipment

#### MR scanners

Data were collected using two different clinical 3 Tesla MRI scanners from two different vendors: A Philips Achieva scanner (Philips, Best, The Netherlands) equipped with a 32 channel RF head array coil and a Siemens Prisma Fit (Siemens, Erlangen, Germany) scanner equipped with a 64 channel RF head array coil. The scanners were located respectively at Glostrup and Blegdamsvej campus of Rigshospitalet, Copenhagen, Denmark; The Philips Achieva scanner was equipped with a 32- and a 64-channel MR Cap (BrainCapMR, Brain Products GmbH, Munich, Germany), which consisted of 63 EEG electrodes, 1 external ECG electrode, 1 ground (AFz), and, 1 reference electrode (FCz). The Siemens Prisma scanner was equipped with a Geodesic EEG System (Electrical Geodesics, Inc., Eugene, OR) with 256 EEG channels, 2 external ECG electrodes and 1 reference electrode (Cz).

Phantom measurements were performed using a spherical daily quality assurance phantom provided by the manufacturers.

#### EEG equipment

BrainCapMR: This device was used on the Phillips Achieva scanner. The system had 64 ring-type sintered nonmagnetic Ag/AgCl electrodes. The EEG cap was selected according to the head size (56, 58 or 60 cm).

Geodesic EEG System MR: This device was used on the Siemens Prisma Fit scanner. The system had 256 channels consisting of sponge based electrodes. The EEG cap was selected according to the head circumference (54–56, 56–58 or, 58–61 cm).

#### Temperature measurement equipment

Temperature measurements in the Philips Achieva and the Siemens Prisma scanner were performed using a four channel Luxtron 3100 fiber optic thermometer (LumaSense Technologies Inc.). In the Phillips scanner, an extra 2-channel Veris MR-compatible monitoring system (MedRad Inc.) was used to monitor additionally the scanner bore and room temperature (and in the last subject also the ear temperature due to failure of one of the Luxtron channels).

### Data acquisition

#### MR scanning protocols

The acquisition parameters for the different acquisition methods are listed in Table 1.

**Table 1:** fMRI acquisition parameters.

Scanner (array coil)	Philips Achieva (32 cha)						Siemens Prisma (64 cha)	
fMRI Method	EPI	EPI	MEPI	MEPI	MB4-EPI	MS-EVI	EPI	MB8-EPI
TR [ms]	3034 (*)	402	3034	376	450 (**)	487(***)	1980	280
TE [ms]	35	35	9.6	9.6	35	35	35	35
Number of Tes	1	1	4	3	1	1	1	1
Slices	37	6	37	6	24	24	30	32
Number of volumes	200	3000	200	3200	1400	895	150	1100
Scan time [min:sec]	10:16	20:00	10:16	20:03	10:36	04:34	10:14	19:11
Voxel size [mm]	3.1	3.1	3.1	3.1	2.88	4	4	4
Slice thickness [mm]	3.1	3.1	3.1	3.1	3.3	4	4	4
Slice gap [mm]	0.31	0.31	0.31	0.31	0.7	0	1	1
Matrix size	76x73	76x73	76x73	76x73	80x79	64x61	64x64	64x64
Flip angle [degrees]	90	30	90	30	15	30	90	30

* In some cases a TR of 2000 ms was used

** In one case a TR of 381 ms was use

*** In some cases a TR of 300 ms was used.

Cha = Channel, TR = Repetition time, TE = Echo time (minimum TE in case of MEPI), EPI = Echo planar imaging, MEPI = Multi echo planar imaging, MB4-EPI = Multiband-4 echo planar imaging, MS-EVI = Multi-slab echo volumar imaging, MB8EPI = Multiband-8 echo planar imaging.

Conventional whole brain EPI data with a repetition time (TR) of 2 or 3 s were acquired on both scanners. On the *Philips Achieva scanner*, additional data were acquired using partial brain EPI at shorter TR (402 ms), and using whole brain and partial brain multi-echo EPI (MEPI) with 3.2-fold SENSE acceleration, a minimum TE of 9.6 ms, an echo time spacing of 17 ms and TRs of 3034 and 376 ms, respectively. Multi-band (simultaneous multi-slice) EPI data were acquired with 4-fold acceleration (MB4-EPI) using a pulse sequence developed by GyroTools LLC (Zürich, Switzerland). Data reconstruction was performed offline. The initial version of the MB-EPI pulse sequence suffered significant temporal instability and artefactual inter-slice correlations as a result of RF pulse instability that was resolved with an update of the pulse sequence software in the middle of the study. This instability prevented removal of gradient artifacts in the initial data. Sequential multi-slab echo-volumar imaging (MS-EVI) data were acquired using a custom designed implementation by GyroTools LLC (Zürich, Switzerland) based on the method described in Posse et al., 2012 [13]. Sinc-modulated RF pulses with high time-bandwidth product were used for slab selection. The MS-EVI pulse sequence employed 3.2x2 fold 2D-SENSE acceleration along k_y_ and k_z_. Image reconstruction was performed online. On the *Siemens Prisma scanner*, additional data were acquired using a multi-band EPI sequence with 8-fold acceleration (MB8-EPI) and online reconstruction developed by the Center for Magnetic Resonance Research, University of Minnesota [18]. Small timing errors in the initial versions of the EPI and MB-EPI pulse sequences that changed the TR were resolved with an update of the pulse sequence software during the second half of the study. This instability prevented removal of gradient artifacts in the initial data.

**Fig 1:**
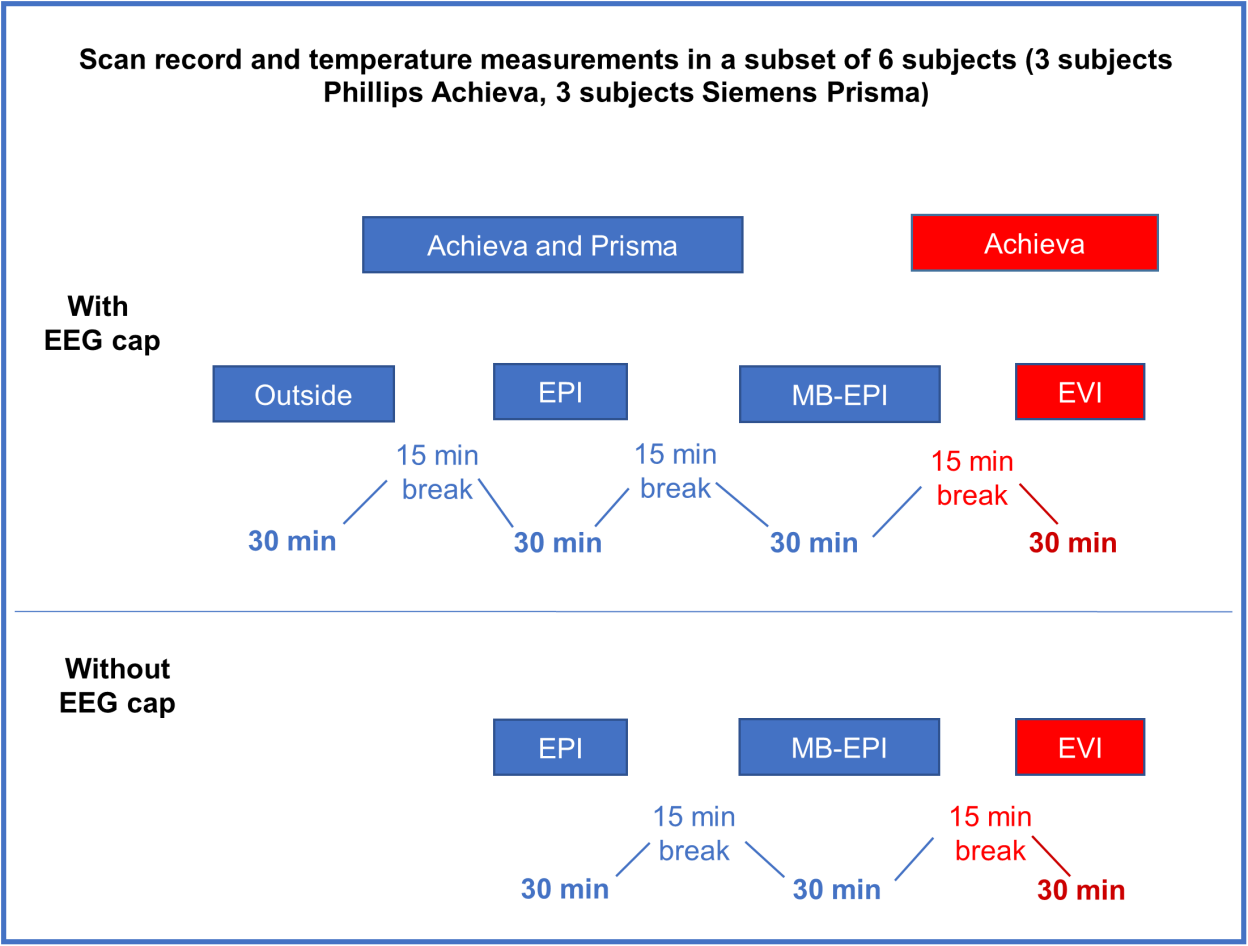
Flowchart of the scan protocol during the systematically temperature measurements.

In the epilepsy patients and the first 5 healthy controls the scanning time for the different pulse sequences varied. In the last 6 subjects (3 in the Philips Achieva scanner and 3 in the Siemens Prisma scanner), all available pulse sequences were applied for 30 minutes (Fig 1). The maximum scan durations and minimum TRs of MB-EPI and MS-EVI were software limited by the operating systems of the scanners to account for gradient coil heating limits and data rate constraints, which were scanner specific.

In the epilepsy patients and the first 5 healthy controls resting-state scans were acquired using a scan duration of 5, 10 or 20 minutes, depending on the fMRI data acquisition method and scanner type. In the last 6 subjects, all sequences were applied for 30 minutes (Fig 1). Subjects were instructed to keep their eyes open, clear their mind and relax without moving the gaze.

#### EEG

In phantom scans the EEG cap was placed on top of the phantom with electrodes touching the surface of the phantom.

BrainCapMR (Phillips Achieva): The skin was prepared using disinfectant and Nuprep gel. The cap was placed according to the international 10–20 system. Two additional channels recorded eye movements (EOG, placed on the forehead) and electrocardiogram (ECG, placed at the subject’s back at the level of the heart just left of the spine). The reference channel was placed at the vertex. Conductive gel was filled in each of the electrodes after positioning the cap, and added when necessary to keep impedance below 20 kΩ. The EEG cap was kept in place by a net. The cables from the cap were run through a small hole in the back of the head coil and stretched and fixed using sandbags to avoid loops. Additional ECG was recorded with 4 leads, 3 electrodes placed along the left side of the sternum and 1 electrode placed in the axillary line. Data were acquired with 5 kHz sampling rate synchronized to the scanner clock frequency.

Geodesic EEG System MR (Siemens Prisma Fit scanner): The skin was disinfected using a non-alcoholic disinfectant. The reference electrode was placed at the vertex. The 256-channel cap was prepared with an electrolyte/shampoo solution. The cap was then applied and adjusted to ensure that selected electrodes were placed at predetermined reference points. The EEG cap was kept in place by a net, and on top of this a swim cap was placed to prevent evaporation of the preparation solution. The impedance was measured, and kept below 50 kΩ. Two ECG electrodes were placed on the left side of the sternum, one at the 3th-4th intercostal space and one at the 6th-7th intercostal space. The cables from the cap were run along the side of the head downwards towards the feet inside the head coil. Data were acquired with 1 kHz sampling rate and synchronized to the scanner clock frequency.

#### Temperature measurements

In phantom scans temperature sensors were distributed across the surface of the phantom and placed underneath EEG-electrodes. In vivo temperature measurements were performed in all subjects, but not all sequences were systematically used in the first 5 controls and in the patients. In the last 6 controls, 3 in the Achieva scanner and 3 in the Prisma scanner, temperature was measured systematically using all available pulse sequences with and without the EEG cap (Fig 1). Sensors were placed underneath EEG-electrodes (see electrode display in supporting information, S1 Fig) on the forehead (with free heat exchange with the surroundings), the left or the right ear, the neck (with limited heat exchange with the surroundings), and on the scanner bore. In two subjects room temperature, but not bore temperature were measured. In measurements performed without the EEG cap the temperature probes were fixed to the subject’s scalp at the same points as with the EEG cap, using sensitive skin tape.

### Data analysis

#### Analysis of signal-to-noise

The signal-to-noise ratio in fMRI scans was computed by measuring the average signal intensity in a circular ROI in occipital cortex, subtracting the average of the signal intensity in 4 circular reference regions outside of the brain in the 4 corners of the image, and scaling the result with the average of the standard deviation of the noise in these 4 reference regions. Differences in SNR between scans with and without the EEG cap on were assessed using a two-tailed heteroscedastic t-test.

#### Analysis of head movement

Head movement in the fMRI data was analyzed using the MCFLIRT toolbox in the FSL software package (http://fsl.fmrib.ox.ac.uk/fsl/fslwiki/)) and reported as relative (dynamic-to-dynamic) and absolute (relative to the middle dynamical image) motion parameters.

#### EEG preprocessing

Due to the timing errors in the EPI and MB-EPI pulse sequences on the Prisma scanner and in the EGI acquisition software, which resulted in failure of gradient artefact removal, it was not possible to assess the data quality of the high-density 256 channel EEG array implemented in the Siemens Prisma scanner.

The EEG data acquired with the 64-channel BrainCapMR equipment on the Philips Achieva scanner were preprocessed using BrainAnalyzer 2.0 (Brain Products GmbH, Gilching, Germany). These steps included gradient artifact removal using the sliding window approach with 21 averages [19], down-sampling from 5kHz to 500 Hz and correction of BCG using the sliding window approach. Data with evident noise after visual inspection were additionally run through an ICA where noise components were removed before a back-projection was applied (BrainAnalyzer 2.0).

#### Evaluation of EEG recordings and scoring of data quality

A board certified clinical neurophysiologist with more than 10 years of experience (SB) visually evaluated the EEG recordings after artifact removal. The neurophysiologist was blinded to which MR sequence was used when the individual EEG’s were recorded. Signals were inspected both in sensor-space and in source-space using BESA Research software [20]. The source montages reproduces the signals in 19 brain regions in each hemisphere [21].

Data quality was scored, using the following criteria:

- good = no or minimal amount of artifact, that does not interfere at all with the visual evaluation / interpretation of > 80% of the recording (score 1).
- acceptable = significant amount of artifacts, yet good enough to distinguish artifacts from background activity and epileptiform discharges (if any) in 40–80% of the recording (score 2).
- poor = the recording is unreadable / less than 40% of the recording is relatively free of artifacts (score 3).

#### Statistical analysis

Data were described with mean and standard deviation for continuous data and sample size and percentage for categorical data.

A logistic mixed model was used to assess the influence of the fMRI type (high-speed vs. conventional), relative and absolute motion on the ratings (poor vs. acceptable and good). The model was adjusted for the disease status of the subject (healthy vs. epilepsy disease). A random intercept was used to account for a potential correlation in the ratings between scans corresponding to the same individual.

Results are reported using odds ratios and their 95% confidence interval. An odds ratio is a measure of association between an exposure (e.g. motion) and the rating. It is the ratio between the odds of occurrence of the outcome given one unit increase for the exposure (e.g. motion increased by one mm) compared to the odds of a reference level [22]. The typical scale for absolute motion is mm whereas for relative motion it is 0.1 mm. Therefore, the odds ratios for these variables are reported in units one mm increase and 0.1 mm increase, respectively. Analyses were performed with R software, version 3.2.3 [23].

## Results

### Temperature measurements

#### Phantom scans

Temperature increases during standard EPI scans of up to 20 min duration did not exceed 0.1°C/min in the Philips Achieva. Temperature increases during MB-EPI and MS-EVI scans measured in the Philips Achieva and Siemens Prisma scanners were in the same range with a maximum of 0.1°C/min. The dependence of temperature increases on SAR of the MB-EPI scanning protocol was further investigated on the Philips Achieva scanner by increasing the flip angle to 256° (8.5 times larger than the flip angle used in vivo) with a SAR value of 51% (Table 2). A 33-minute scan time resulted in a temperature increase of 0.06°C/min.

**Table 2:** Temperature measurements and SAR values during MB-EPI sequences in concurrent EEG and MRI in the Phillips Achieva scanner demonstrating the impact of increased flip angle.

Subject	Sequence	Flip angle (degree)	Scan Duration [min]	SAR [%] (Mean/SD)		SAR [%] (Max)	ΔT [°C] (Mean/SD)		ΔT [°C] (Max)
Phantom (n = 1)	MB-EPI	256	33.00	51	0	51	0.8	0.3	1.1
Phantoms (n = 2)	MB-EPI	120	20.0/30.0	35	6	39	0.5	0	0.5
Control (n = 1)	MB-EPI	120	4.27	31	0	31	0.8	0.5	1.1
Control + patients (n = 4)	MB-EPI	15	10.0/20.0	18	2	21	0.2	0.3	0.8

Measurements with increased flip angle are in *italics*. Temperature measurements from all probes were combined.

ΔT: Absolute temperature change.

#### In-vivo scans

**Fig 2:**
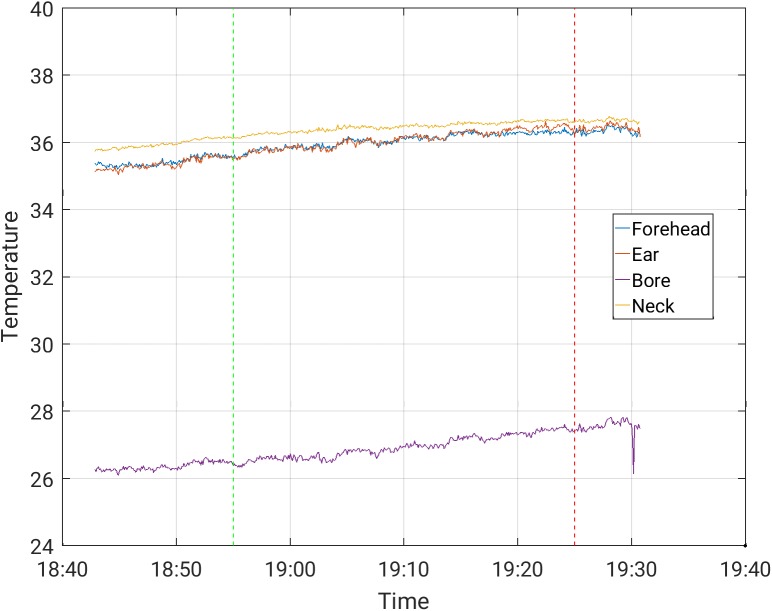
Temperature measurement during a 30-minute EPI sequence with EEG cap on, in the Siemens Prisma scanner.

**Fig 3:**
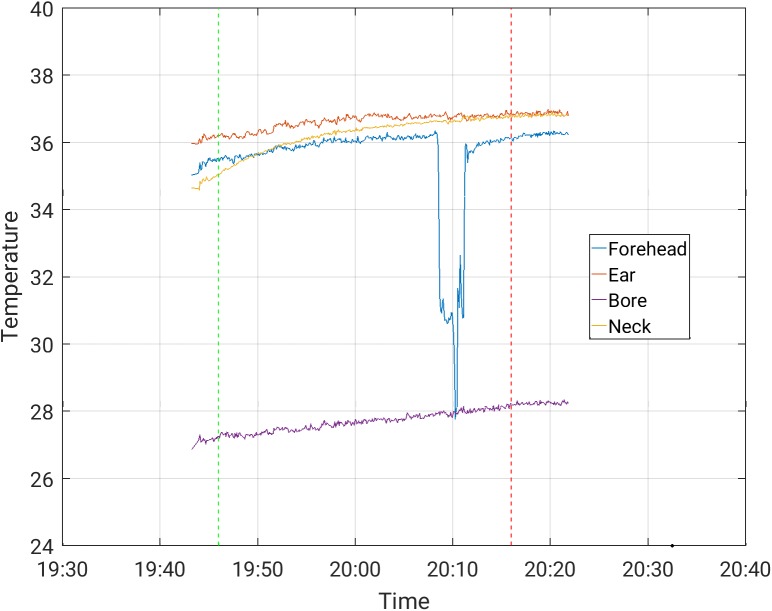
Temperature measurement during a 30-minute MBEPI sequence with EEG cap on, in the Siemens Prisma scanner (forehead temperature channel disconnected at the dip).

**Fig 4:**
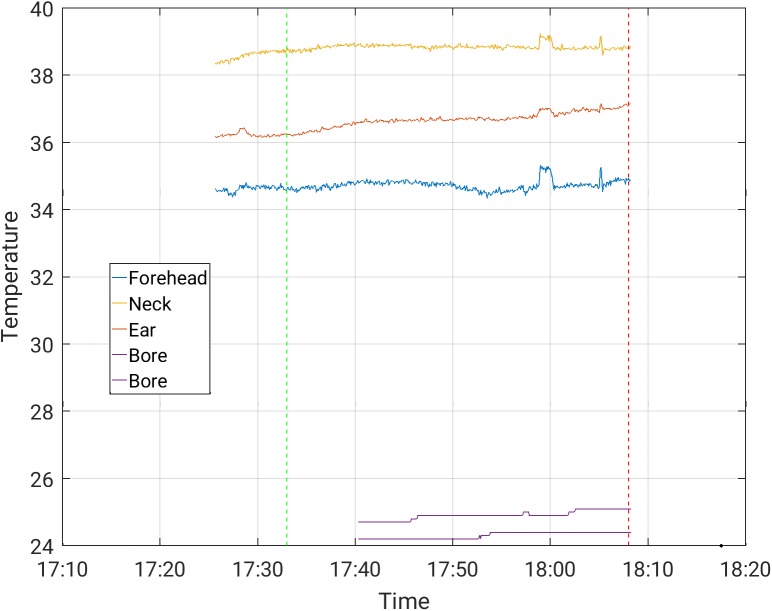
Temperature measurement during a 30-minute EPI sequence with EEG cap on, in the Phillips Achieva scanner.

**Fig 5:**
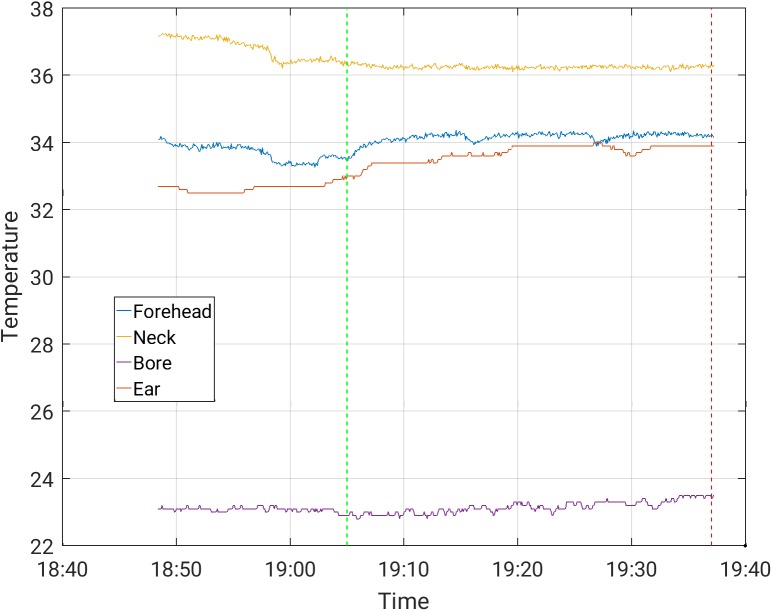
Temperature measurement during a 30-minute MBEPI sequence with EEG cap on, in the Phillips Achieva scanner.

**Fig 6:**
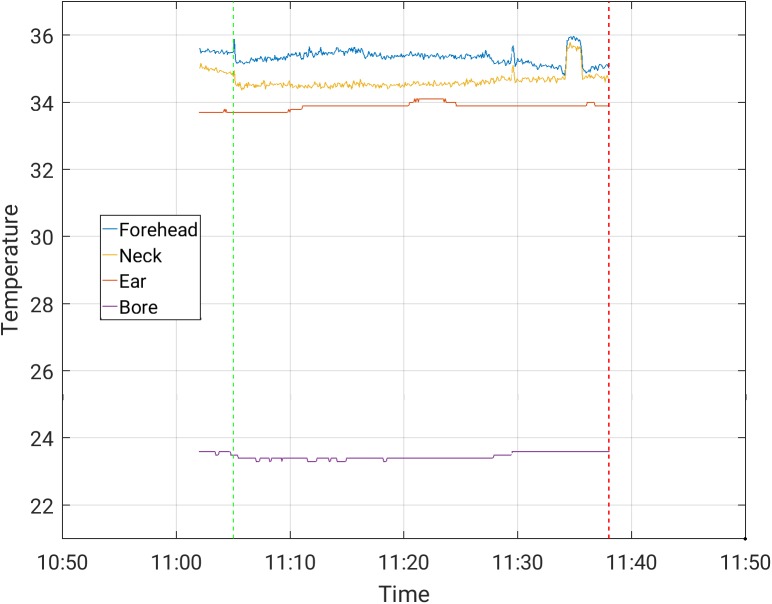
Temperature measurement during a 30-minute EVI sequence with EEG cap on, in the Phillips Achieva scanner.

The increases in temperature while the subjects were scanned with and without the EEG cap (Table 3) were comparable. The maximal temperature increase, across channels, observed in scans where the temperature was stable prior to the beginning of the scanning, was 0.50°C in a 30-minute scan period for the Siemens Prisma scanner, and 1.0°C in a 30-minute scan period for the Phillips Achieva scanner, for all protocols. These highest increases were measured during MB-EPI on both the Philips Achieva and Siemens Prisma scanners. Representative temperature curves where artifacts are minor are shown in Figs 2–6. Data from which the curves were constructed can be found in supporting information S1-S5 data.

**Table 3:** Summary of temperature measurements.

Scanner (head RF array coil)	Siemens Prisma (64 ch) (N = 3)			Phillips Achieva (32 ch) (N = 3)		
**Temperature Change [°C]**	Frontal	Occipital	Temporal	Frontal	Occipital	Temporal
Mean outside	1.3	1.5	1.3	1.2	0.0	2.2
Mean inside w/o scanning	0.1	0.6	0.9	0.1	1.5	0.4
Mean EPI with cap	0.2	0.4	0.6	0.2	0.1	0.8
Mean MB-EPI with cap	0.1	0.6	0.5	0.5	0.4	0.9
Mean MS-EVI with cap				0.1	0.2	1.2
Mean EPI w/o cap	0.2	1.9	1.4	-0.1	0.3	
Mean MB-EPI w/o cap	0.5	1.7	0.7	-0.1	1.2	0.2
Mean MS-EVI w/o cap				0.2	0.2	
**Temperature Gradient [°C/min]**						
Mean outside	0.041	0.048	0.041	0.038	0.000	0.073
Mean inside w/o scanning	0.010	0.057	0.087	0.005	0.150	0.035
Mean EPI with cap	0.007	0.013	0.019	0.005	0.002	0.025
Mean MB-EPI with cap	0.003	0.018	0.016	0.016	0.013	0.028
Mean MS-EVI with cap				0.002	0.006	0.036
Mean EPI w/o cap	0.008	0.058	0.042	-0.002	0.009	
Mean MB-EPI w/o cap	0.017	0.058	0.022	-0.003	0.036	0.006
Mean MS-EVI w/o cap				0.007	0.006	

The average head SAR values for a standard 70 kg person in the Phillips Achieva scanner without cap for EPI, MB-EPI and MS-EVI were 25%, 17% and 14%, respectively (Table 4). The mean values for these sequences in vivo with the 64 channel EEG cap on were similar: EPI 24% (± 1), MB-EPI 18% (± 2) and MS-EVI 13% (± 3). These SAR values corresponds to TR’s of 3034 ms in the EPI, 450 ms in the MB-EPI and 487 ms in the MS-EVI (Table 1). In the Siemens Prisma scanner the mean SAR values showed a considerable increase with the 256 channel EEG cap on: EPI with cap 19% (± 3), MB-EPI with cap 16% (± 3), EPI without cap 8% (± 0), MB-EPI without cap 7% (± 1) (Table 4). The corresponding TR in the EPI were 1980 ms and in the MB-EPI 280 ms (Table 1).

**Table 4:** Summary of SAR values.

			SAR [%]		
Scanner	EEG System	Sequence / EEG cap	Mean	SD	Max
Philips Achieva	Brain Products 64 channel	EPI_with_cap	24	1	25
		EPI_without_cap	25		
		MB-EPI_with_cap	18	2	21
		MB-EPI_without_cap	17		
		MS-EVI_with_cap	13	3	17
		MS-EVI_without_cap	14		
Siemens Prisma	EGI 256 channel	EPI_with_cap	19	3	23
		EPI_without_cap	8	0	8
		MB-EPI_with_cap	16	3	18
		MB-EPI_without_cap	7	1	8

The dependence of temperature increase on SAR during MB-EPI scans was further investigated on the Philips Achieva scanner in a healthy control using a 120° flip angle (4 times larger than in the standard protocol) and a 4:27 min scan duration, which resulted in a maximum temperature increase of 0.26°C/min at an SAR of 31% (Table 2). This SAR is still considerably smaller than that of the TSE sequence reported in [24].

Additionally, temperature measurements outside of the scanner and while positioning the subjects inside the scanner prior to scanning (Table 3) show that temperature could increase irrespectively of scanning taking place. With a few exceptions, this increase inside the scanner prior to scanning was more pronounced than the temperature increases during scanning, indicating that physiological and environmental factors dominated the measured temperature increases. Temperature increases with the cap on were more pronounced outside of the MR scanner than inside the scanner during scanning (Table 3). As temperature changes, may be influenced by several factors, including MR-scanning, physiological and environmental changes (e.g. bore temperature) these factors were taken into account when analyzing the temperature changes. This was primarily done by analyzing temperature changes in the minutes before scanning was started.

Subjective temperature sensations and comfort of wearing the EEG cap were additionally assessed by asking subjects repeatedly to report on any heating sensations during the scanning. None of the subjects reported an electrode related heating sensation. The application of the EEG caps was well tolerated and the comfort of wearing the cap inside the MR scanner was comparable for both scanners. The subjects reported a similar degree of discomfort when the RF coil was closed, which was primarily a slightly claustrophobic sensation. All subjects reported some pressure from the electrodes after a scanning session lasting more than 30 minutes.

### Signal-to-noise measurement

In the resting state fMRI scans using the 256 channel EEG system on the Siemens Prisma scanner, the SNR values averaged across all scans showed a trend level decrease (p < 0.063) when subjects were wearing the EEG cap (159+/-16) compared to when not wearing the EEG cap (171+/-19). Using the 64 channel EEG system on the Phillips Achieva scanner no such difference was found (p > 0.90). The cortical SNR when the subjects were not wearing the EEG cap (140+/-37) was very similar to when they were wearing the cap (138+/-38).

### EEG data quality

**Fig 7:**
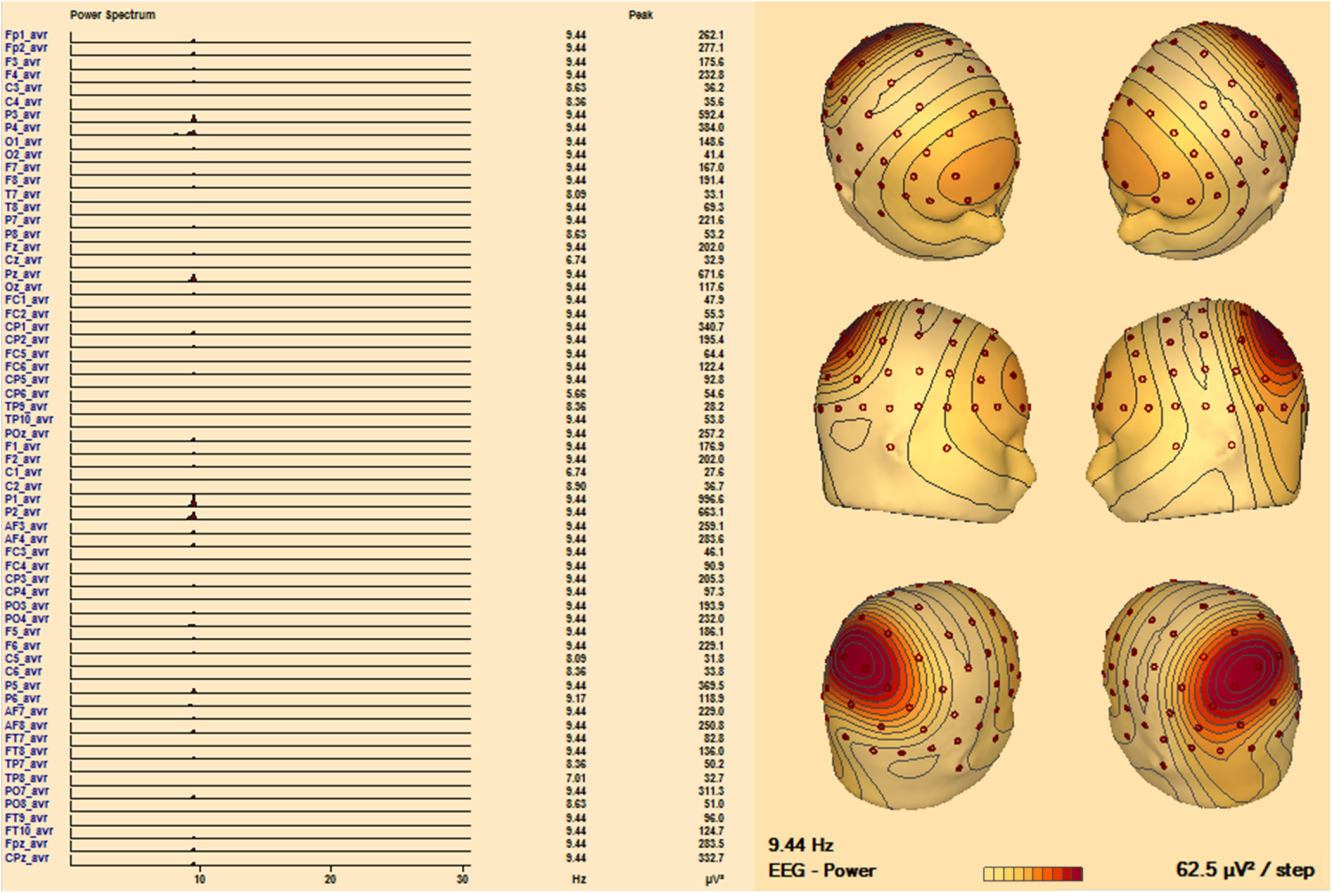
Posterior dominant (alpha) rhythm in an EEG recorded in the MR scanner, during a high-speed sequence. **Left:** Power Spectrum at EEG electrodes demonstrates a clear peak at 9.44 Hz, at the occipital electrodes. **Right:** power-map of alpha activity showing its 3D distribution.

In the Philips Achieva scanner using the 64 channel EEG array, data quality was strongly related to electrode impedance. An impedance level below 20 kΩ was required for optimum data quality. The posterior alpha rhythm in the resting state data was clearly detectable, and located in the occipital area (Fig 7). The presence of bilateral, symmetric rhythmic activity in the range of 8–12 Hz (Shaun et al., 2013), in the posterior (occipito-parietal) region in all patients demonstrated that the normal ongoing EEG (background) activity was not distorted by the data processing [25].The 64 channel EEG data quality inside the scanner during scanning was significantly affected by the presence of residual BCG artifacts and a loss of nuances/information in the EEG compared with data acquired outside the scanner.

Descriptive data for the EEG quality and motion regarding high-speed and conventional MR sequences is shown in Table 5. Visual analysis of EEG quality by a trained expert could not demonstrate a significant difference in data quality between conventional EPI and high-speed fMRI sequences (MS-EVI and MB-EPI) (p = 0.78). There was no evidence that EEG signal quality was affected by absolute (p = 0.49) or relative motion (p = 0.36).

**Table 5:** EEG quality using conventional versus high-speed MR sequences and dependence on head motion.

	Conventional (EPI and MEPI)	High-speed (MS-EVI and MB-EPI)	Total
Number of scans	30	20	50
**EEG quality** (number (%) of poor ratings)	18 (60%)	11 (55%)	29 (58%)
**Motion relative** (mm)	0.1 (0.1)	0.2 (0.1) †	
**Motion absolute** (mm)	1.4 (1.1)	0.9 (0.6) †	

Relative and absolute motion are reported using mean and standard deviation.

EPI = Echo planar imaging. MEPI = Multi-echo planar imaging. MS-EVI = Multi-slab echo volumar imaging. MB-EPI = Multi-band echo planar imaging.

†Data from 4 scans missing.

## Discussion

The present study demonstrates that concurrent EEG and fMRI can be performed safely across the wide range of fMRI acquisition methods and the body coils used in this study. Although temperature rises of up to 1° C underneath some of the electrodes were measured in a 30-minute scan period, they were on average of such small magnitude that RF induced heating was not a limiting factor for the experimental settings in this study. Temperature drifts of a similar and even larger magnitude were frequently noted even without scanning. In the Prisma scanner a significant increase in SAR was observed when the subjects wear the EEG cap. The tight fit of the EEG cap inside the 64-channel head coil of the Prisma scanner in some of the subjects was a concern, since it could introduce the possibility of an incomplete mechanical closure of the coil, leading to possible coil malfunction and mistuning with the potential for increases in local SAR independent of the presence of the EEG electrodes. It may also restrict circulation, which could cause the sensation of heating. These may have contributed to the localized heating reported by one subject in the Prisma scanner during preliminary setup of the protocol. The maximum SAR values in the present studies, even for scans exploring the safety margins, were well below those reported in previous studies using Fast Spin Echo and Turbo Spin Echo pulse sequences that can reach up to 100% of the FDA approved SAR limits [26] [24]. Although gradient coil heating due to gradient switching limited data acquisition rates and maximum scan times, the present study allows to estimate safe limits for longer scan times as temperature increases are usually strongest at the beginning of the scan. Despite these still encouraging results one should not forget that placing electrodes and wires into high field scanners during high-speed fMRI requires constant attention for the safety and comfort of the subjects. There is potential for local temperatures to increase rapidly, if basic safety requirements such as adequate air circulation, proper closure of the coil, and avoidance of wire loops are not met.

The EEG data quality using the 64 channel EEG system during MR scanning was considerably degraded compared to EEG data measured outside of the scanner. However, EEG data quality was not significantly different between the conventional and the fast sequences. The quality of the EEG data was visually evaluated by a trained expert. This could be relevant, since epileptiform discharges are also identified this way (visual evaluation by trained experts). However, this visual approach is subjective, and thus represents a potential bias, although the EEG expert was blinded to the type of MR sequence when evaluating EEG quality. We consider this a limitation of the study.

Due to initial timing errors in the EPI and MB-EPI pulse sequences and in the EGI software, which resulted in failure of gradient artefact removal, we have not been able to assess the data quality of the high-density 256 channel EEG array implemented in the Siemens Prisma scanner. This comparison will be performed in a future study. There is now compelling evidence that detection and identification of spikes is significantly improved using electric source imaging with high-density electrode arrays (HD-EEG), with extended coverage including the face and neck areas [27] [28]. More than 90% of the spikes identified by HD-EEG were missed by the classical 10–20 electrode array [29]. Voltage maps derived from HD-EEG recording outside the MR scanner can serve as templates for algorithms searching for spikes in the EEGs recorded inside the MR-scanner [30]. A recent study described sensitivity advantages of concurrent HD-EEG-fMRI for mapping the epileptic networks [31]. However, other studies showed considerable decreases in the signal to noise ratio in fMRI due to susceptibility inhomogeneity, although no significant changes in task-based activation and resting state connectivity were detected [9] [10] [32]. This is consistent with our observation of trend level, decreases in cortical SNR due to the presence of the 256 channel EEG cap, in contrast to the 64 channel cap, which showed no significant difference in SNR.

Another possible source of artifacts is RF noise that may be transmitted through the EEG leads. Further, EEG leads and electrodes may distort the transmit RF field (B1+) and the receive RF field (B1-) profiles, which causes regional flip angle variations and spatial variations in sensitivity, resulting in regional variation of fMRI signal intensity and degradation of the SNR [10]. However, fMRI at the typical voxel sizes used in clinical studies is dominated by physiological noise and as stated above, most previous studies have reported that the sensitivity of task-based and resting state fMRI is not significantly affected by the presence of the EEG cap [32] [10] [9].

Concurrent EEG-fMRI is a tool that is of considerable interest for many aspect of neuroscience, e.g., evoked potentials, sleep and epilepsy. Although the electrodes put some pressure on the skin, and especially for the electrodes the subjects were lying on, the use of the EEG cap in our study was well tolerated and did not limit the duration of the studies. Among the potential clinical use of concurrent EEG-fMRI should be mentioned selected patients with pharmacoresistant epilepsy where it is well documented that seizure freedom can be obtained after surgical removal of the epileptogenic tissue [1]. However, localizing the epileptogenic zone is often complex and challenging as both epileptic discharge and symptoms may spread during the attack. Therefore, pre-surgical evaluation of structure and function to localize the epileptogenic zone and adjacent eloquent cortex in these patients becomes a major goal for rendering as many patients as possible seizure free without neurological deficits. The approach is multimodal and in some patients invasive EEG is necessary with electrodes placed on the brain surface or inside the brain. In this context, concurrent acquisition of fMRI and EEG has emerged as a new potential tool for localizing the epileptogenic zone.

## Conclusions

This study demonstrates that high-density EEG can be safely implemented in conjunction with high-speed fMRI. Despite these encouraging results one should not forget that placing electrodes and wires into high field scanners that are equipped with high-power RF amplifiers requires constant attention for the safety and comfort of the subjects. We recommend performing temperature measurements with any new pulse sequence. High-speed fMRI does not adversely affect EEG data quality as visually evaluated by a trained expert. However, the deterioration of the EEG quality due to residual ballistocardiographic artifacts remains a significant constraint for routine clinical applications of concurrent EEG-fMRI.

## Supporting information

S1 Fig EEG cap with the respective temperature probes. Electrode display in the 256-channel (left) and the 64-channel (right). The temperature probes were placed underneath the electrodes marked with the red circle, or underneath one of the neighboring electrodes. The ear temperature probe was placed on the left or the right side.
(TIF)

S1 Data From which Fig 2 was constructed. (CSV)

S2 Data From which Fig 3 was constructed. (CSV)

S3 Data From which Fig 4 was constructed. (CSV)

S4 Data From which Fig 5 was constructed. (CSV)

S5 Data From which Fig 6 was constructed. (CSV)

## Abbreviations

BCG: Ballistocardiographic
BOLD: Blood oxygenation level dependent MR response
EEG: Electroencephalography
EEG-fMRI: Concomitant EEG and fMRI
EGI: Electrical Geodesics, Inc
EPI: Echo planar imaging
fMRI: functional magnetic resonance imaging
ICA: Independent component analysis
MB-EPI: Multi-band EPI
MEPI: Multi-echo EPI
MS-EVI: Multi-slab echo volumar imaging
MR: Magnetic resonance
MREG: MR-encephalography
RF: Radiofrequency
rsfMRI: resting state fMRI
SAR: Specific absorption rate
TE: Time to echo
TR: Time to repetition
