# Supplementary figures and images for: Safety and EEG data quality of concurrent high-density EEG and high-speed fMRI at 3 Tesla
Source: PLoS One. 2017 May 26;12(5):e0178409. doi: 10.1371/journal.pone.0178409 (PMC5446172; doi:10.1371/journal.pone.0178409)

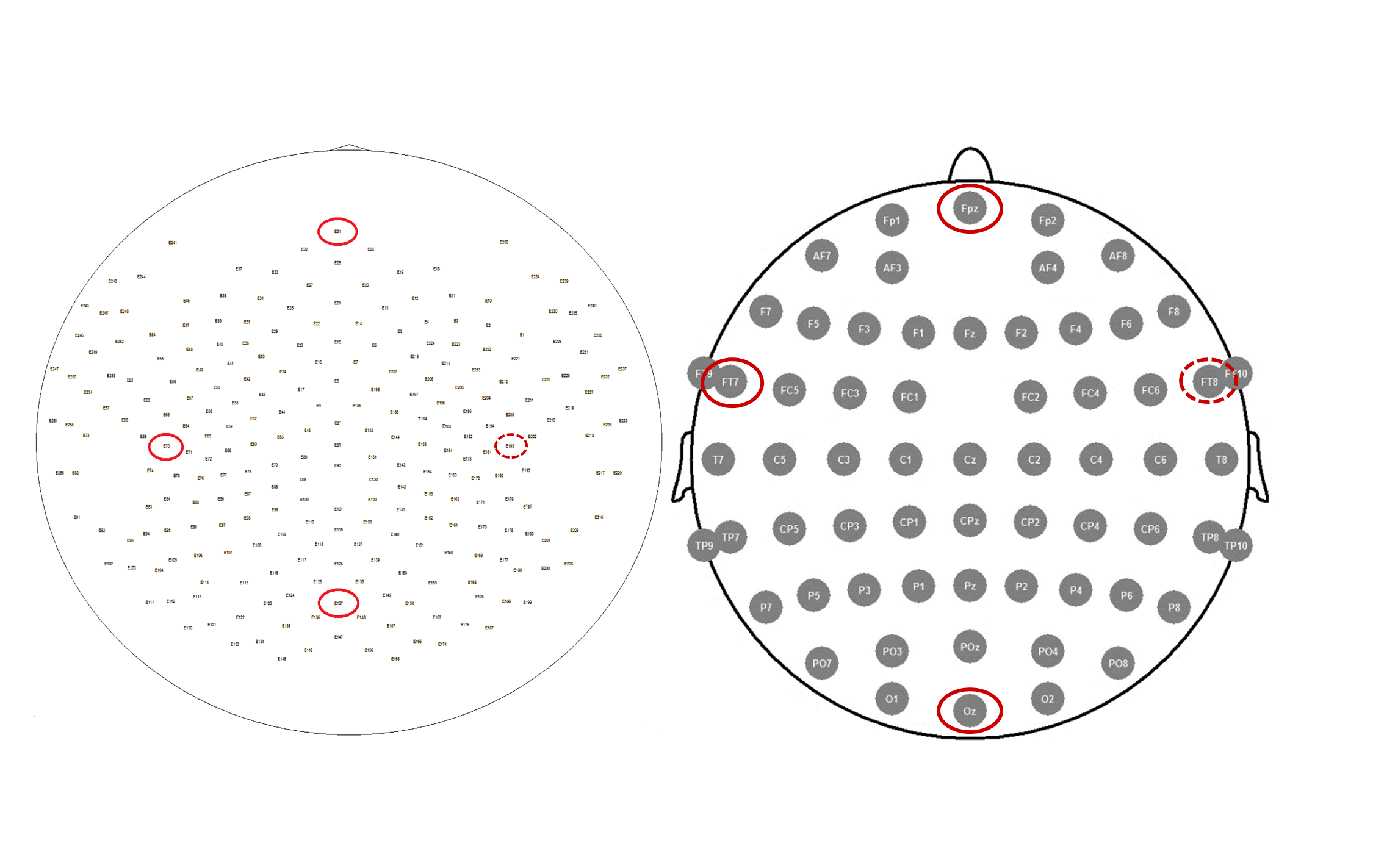

Supplement: S1 Fig — Electrode display in the 256-channel (left) and the 64-channel (right). The temperature probes were placed underneath the electrodes marked with the red circle, or underneath one of the neighboring electrodes. The ear temperature probe was placed on the left or the right side. (TIF) [file pone.0178409.s001.tif]
